# The Contributions of Updating in Working Memory Sub-Processes for Sight-Reading Music Beyond Age and Practice Effects

**DOI:** 10.3389/fpsyg.2019.01080

**Published:** 2019-05-15

**Authors:** Laura Herrero, Nuria Carriedo

**Affiliations:** ^1^Facultad de Educación y Salud, Universidad Camilo José Cela, Madrid, Spain; ^2^Departamento de Psicología Evolutiva y de la Educación, Facultad de Psicología, Universidad Nacional de Educación a Distancia (UNED), Madrid, Spain

**Keywords:** music sight reading, updating in working memory, retrieval, transformation, substitution, practice

## Abstract

Music sight reading (SR), has been described as a complex task which involves the simultaneous reading of new non-rehearsed material and performance. Although practice related skill have revealed as the most significant predictor of SR, working memory (WM) processes have shown its relevance in the study of individual differences in SR. We aimed to determine how the updating in WM sub-processes of retrieval/transformation and substitution, could differentially contribute to SR when the effects of age and practice were controlled, and according to the difficulty of the SR tasks and the different indexes of performance measured (SR error, tempo maintenance, rhythmic accuracy, pitch accuracy, articulation accuracy and expressiveness). 131 music students of different ages and levels of instrument knowledge participated in the study. The results showed that whereas the efficiency in the retrieval/transformation sub-processes contributed to SR regardless of the difficulty of the SR tasks, the substitution sub-process also contributed to performance at sight but only in low demanding SR tasks. The results also showed all the updating sub-processes were engaged in SR regarding the proportion of error and rhythmic accuracy. However, both expressiveness and tempo maintenance seemed to be uniquely driven by efficiency in the retrieval/transformation sub-processes, whereas articulation accuracy relied on the efficiency to suppress irrelevant information from WM.

## Introduction

Music sight reading (SR), has been considered as one of the five basic abilities for all musicians (McPherson and Thompson, 1998). SR has been described as a complex transcription task involving the simultaneous reading of new non-rehearsed material and performance (Sloboda, 1982; Thompson, 1987; Waters et al., 1997, 1998). Contrary to rehearsed music, which offers certain automation of performance though deliberate practice, SR would be considered as a novel task. In addition, SR could be considered as a kind of multitasking performance because it requires at least: (a) the processing of visual information linked to reading, including decoding and understanding the score; (b) the motor control linked to performance, including fine motor control and musicality; and (c) the processing of auditory information linked to the adjustment of performance to the printed material, all in real time. Thus, due to the fact that SR requires the simultaneous execution of various tasks in a brief period of time it could be linked to executive functioning (Oswald et al., 2007).

In this context, our main objective was to analyze the role of updating information in working memory (WM) executive function in SR performance, in string and wind musicians of different ages and levels of instrument knowledge.

Updating information in WM is one of the functions related to executive control (Miyake et al., 2000). It was initially defined by Morris and Jones (1990), as the mechanism responsible for the replacement of WM content that is no longer relevant in the ongoing task for new novel material, and of the adjustment of remaining material to the incoming new material. Updating information in WM has been highly related to WM capacity (WMC) (Miyake et al., 2000; St Clair-Thompson and Gathercole, 2006; Belacchi et al., 2010). However, Ecker et al. (2010) – using structural equation models (SEM) – concluded that WMC and updating could be distinguishable in terms of their underlined sub-processes, specifically: (a) retrieval of the relevant information from Long Term Memory (LTM); (b) transformation of information in WM; and (c) substitution of information in WM. According to Ecker et al. (2010), the substitution sub-process may be the only sub-process specific for updating, whereas both retrieval and transformation could be a shared source of variance between WMC and updating. Thus, substitution would be the base for differentiating between updating and WMC. Other authors have also empirically differentiated between the sub-processes of retrieval/transformation and substitution (Chiappe et al., 2000; De Beni and Palladino, 2004; Carretti et al., 2005, 2009; Passolunghi and Pazzaglia, 2005; Lechuga et al., 2006; Carriedo et al., 2016), showing different developmental patterns (Lechuga et al., 2006; Carriedo et al., 2016), and also different predictive values in the study of individual differences in some complex skills such as reading comprehension (Chiappe et al., 2000; Carretti et al., 2009) or math problem solving (Passolunghi and Pazzaglia, 2005).

Other subsequent study carried out by Ecker et al. (2014), in which the substitution sub-process was analyzed, revealed that the efficient substitution of no longer relevant information could require the continuous shifting between between removal and the encoding. They proposed that the active removal could be critical to allow the WM system to efficiently focus on relevant information. Other authors such as Lavie et al. (2004) suggested that it may be the increase in memory load which could drain the availability of attentional resources to reject no relevant information. Finally, efficiency theories posted the existence of a trade-off effect of the attentional resources available to simultaneously maintain and suppress information in WM (e.g., Just and Carpenter, 1992; Kane and Engle, 2000; Engle, 2002).

To our knowledge, there is no empirical evidence that addresses the role of updating information in WM sub-processes in SR performance. However, some previous studies revealed the involvement of the related WMC in it. Specifically, Kopiez and Lee (2006), focused in expert adult pianists to analyze rhythmic accuracy in SR, considered the most objective and reliable measure of SR execution (e.g., Elliott, 1982; Hodges, 1992; McPherson, 1994; Waters et al., 1997; Drake and Palmer, 2000; Gromko, 2004; Kopiez and Lee, 2008; Hayward and Gromko, 2009; Wurtz et al., 2009; Mishra, 2014). They proposed a dynamic model using five SR tasks of an increasing level of difficulty. These authors considered three different types of predictors of SR: (a) general cognitive skills (WM, short-term music memory, short-term numerical memory, and fluid intelligence); (b) elementary cognitive skills (tapping speed, simple reaction time, trilling speed, and information processing speed); and (c) practice-related skills (practicing solo, sight treading, and auditory representation skills practice). Their results showed that practice was the most significant predictor, but that WM also predicted SR performance. However, its influence changed as the level of difficulty of the SR task did, increasing for the easiest levels and decreasing for the most difficult ones. Thus, Kopiez and Lee (2006) concluded that the predictive value of WM and practice related skills for SR would be dynamic, varying in its significance as a function of the complexity of SR tasks.

In a later study with a similar sample, Kopiez and Lee (2008) found that psycho-motor speed, early acquired expertise, mental speed, and the ability for auditory imagery were significant predictors of SR performance, whereas the influence of WM came near of the standard levels of significance (*p* = 0.06).

These apparent contradictory results of both studies could be associated to methodological differences between them. In their first study Kopiez and Lee (2006) analyzed the relation among the different predictors and SR separately by the level of difficulty of the SR tasks. In their second study (Kopiez and Lee, 2008), these relationships were explored taking together all levels of difficulty. Thus, it could be possible that the type of analysis carried out in the second study overshadowed the existing complex relationship between WM and SR.

Another subsequently study carried out by Meinz and Hambrick (2010) with adult pianists of a wide range of instrument knowledge found that WM could also predict SR efficiency independently from general music performance skills^1^.

Considering all these studies, it may be suggested that (a) practice SR related skills could be considered as one of the main predictors of SR performance (but see for a different account: Mishra, 2014; Zhukov, 2017); (b) WM processes seem to be involved in SR performance both among expert musicians and those with different levels of instrument knowledge; and (c) regardless of effect of practice, WM could play a role in SR performance, both in rhythm accuracy and in global subjective performance, but the influence of WM is complex and probably depends on the type of indexes of SR performance obtained, or on the kind of WM task used (Kopiez and Lee, 2008).

Taking into account this rationale, our main aim was to analyze how the sub-processes of retrieval, transformation and substitution underlying updating in WM were related to SR as a function of the demands on memory load and level of suppression, and to analyze how the efficiency in these updating sub-processes could be a source of individual differences in SR as a function of the difficulty of the SR task and of different indexes of performance.

For this purpose, we used an updating task (De Beni and Palladino, 2004), that provided three different indexes – under variable demands of load and suppression – that would correspond to the components of updating executive function described by Ecker et al. (2010): (a) an index of the retrieval and transformation sub-processes (recall of critical items) more related to WMC measures, and (b) two indexes of the substitution sub-process: same lists intrusions, as an index of suppression of information in WM; and previous lists intrusions, as an index of proactive interference or suppression of irrelevant information retrieved from LTM. In addition, we used two different SR tasks of two levels of difficulty which were measured through separate elements of performance (SR error, tempo maintenance, rhythmic accuracy, pitch accuracy, articulation accuracy, and expressiveness).

We expected a positive correlation between the retrieval/ transformation updating index and SR, and a negative correlation between the substitution updating index and SR, both regardless of difficulty of the SR task when age and practice were controlled for. However, considering the possible existence of a trade-off effect between updating sub-processes of retrieval/transformation and substitution (e.g., Just and Carpenter, 1992; Kane and Engle, 2000; Engle, 2002), their relationship to SR could vary as a function of the demands of memory load and level of suppression of the updating task. Thus: (a) if SR were mainly driven by the simultaneous maintenance and processing of information in WM, then there will be a correlation between SR tasks and updating in WM indexes in high load and low suppression condition; (b) if SR were mainly driven by the inhibition of irrelevant or no longer relevant information, then there will be a correlation between SR tasks and updating in WM indexes in low load and high suppression condition; and (c) if both sub-processes were equally relevant for an efficient SR, then there will be a correlation between SR tasks and updating in WM indexes in high load and high suppression condition.

Regardless of whether SR relies more on the retrieval/ transformation or on the substitution sub-processes, we also expected that the efficiency in both sub-processes would significantly contribute to SR performance, especially in the most demanding SR tasks.

In addition, considering the existence of different elements within the musical structure involved in performance such as tempo, rhythm, pitch, or articulation, we expected that the efficiency in the sub-processes of retrieval/transformation and substitution could differently contribute to the proficiency of participants. First, we expected that those efficient participants in the retrieval/transformation sub-process outperformed less efficient ones in those musical elements more related to expressiveness or fluency such as rhythm and tempo maintenance, due to their increased ability to maintain and process information in WM. Secondly, we expected that those efficient participants in the substitution sub-process would outperform less efficient ones in those musical elements more related to accuracy in the execution, such as pitch and articulation, due to their increased ability to suppress no longer relevant information. Thirdly, we expected a similar contribution of the efficiency in both updating-sub-processes regarding SR error index.”

## Materials and Methods

### Participants

One hundred thirty-one music students that have been receiving music lessons of string or wind instruments participated in this study. Participants were classified into four groups according to age and level of instrument knowledge (see Table 1): (1) elementary-low level, formed by 37 children aged 10–11 (*M* = 10.9, *SD* = 0.44; 26 females, 11 males), that have been receiving string or wind instrument lessons for at least 2 years; (2) elementary-high level, formed by 31 pre-adolescents aged 12–13 (*M* = 13.2, *SD* = 0.48; 25 females, 6 males), that have been receiving string or wind instrument lessons for at least 4 years; (3) intermediate level, formed by 32 adolescents aged 15–16 (*M* = 15.2, *SD* = 0.49; 21 females, 11 males, that have been receiving string or wind instrument lessons for at least 6 years; and (4) superior level, formed by 31 young adults (*M* = 22.3, *SD* = 1.34; 14 females, 17 males), that have been receiving string or wind instrument lessons for at least 12 years.

**Table 1:** Instrumental training of the musicians by level of instrument knowledge (means and standard deviations for individual instruction in years and for current individual practice).

Group	Elementary-low	Elementary-high	Intermediate	Superior
Accumulated individual instruction in years	2.9 (0.32)	4.6 (0.44)	6.8 (0.28)	12.9 (0.46)
Individual instruction in minutes per week	30	45	60	90
Group instruction in hours per week	2	3	5	7
Current individual practice in hours per week	2.46 (1.50)	3.19 (2.03)	7.77 (3.10)	21.23 (8.44)


All the participants have learned to read music and to play simultaneously, through a traditional methodology of musical language teaching (rhythm lecture, intonation and music theory without an instrument) and progressive scales, studies and compositions for the instrument without specific SR training. Parents or guardians of the participants under 18 were informed in writing, and signed the consent of participation of their children in all cases. Young adult participants personally agreed to participate by signing a written informed consent form. Ethical approval was provided by the bioethics committee of Universidad Nacional de Educación a Distancia (UNED).

### Materials

#### Sight Reading Test (SR Test)

Two different pieces from “Sound at Sight Series” (Trinity College of London, 2003a,b, 2004, 2007a,b,c,d,e, 2008, 2009a,b) were selected by instrument specialists for each kind of instrument. This series includes specific pieces for each instrument in 8 difficulty grades developed for SR examination in classical western music style. Pieces were selected as a function of the level of general performance of the participants: grade 3 (elementary-low level), grade 5 (elementary-high level), grade 6 (intermediate level), and grade 8 (superior level). The pieces within each level and instrument had similar difficulties regarding key, tempo, rhythm, melodic range, and leap size. Taking into account that the participants played different string, wood wind and brass wind instrument which involve technical performance differences, key, rhythm, melodic range, or leap size could be different among instruments within a similar level of general difficulty. Thus, the difficulty criterion was the time bar, based on previous research which has been shown that binary patterns and subdivisions are easier than ternary ones both in reproduction tasks (Drake, 1993) and in auditory discrimination ones (Smith and Cuddy, 1989). We choose a 2/4 or 4/4 piece (binary time bar and binary subdivision) such as a binary test for all the levels and instruments. In relation to the participant’s instrument knowledge, we selected the ternary pieces using both time bar and subdivision as criterion, that is, a 3/4 piece (ternary time bar and binary subdivision) for the elementary-low level, a 6/8 or 12/8 piece (binary time bar and ternary subdivision) for the elementary-high level, and a 6/8, 12/8, 3/8, or 9/8 (binary or ternary time bar and ternary subdivision) for the intermediate and superior levels.

In order to obtain a complete index of performance at sight, we selected as dependent variables different measures of performance: tempo maintenance, measured as the ability to maintain a fixed tempo during performance with the exception of tempo changes indicated in the score; rhythmic accuracy, measured as the correct proportion between notes and their corresponding rests, which allowed us to control not only the first note in a beat, but also all the notes or rests written in the score; pitch accuracy, measured as the correct tuning; articulation accuracy, measured as the correct performance of articulation signs. We also included an index of expressiveness, measured as general phrasing and musicality, and in the adjustment to dynamics, tempo changes or either expressive indications printed in the score. All these variables were blindly and independently evaluated from 0 to 10 by two expert musicians. They were professional musicians with a full education degree in classical western music (piano and viola), which include 10 years of instrument training together with solfeggio, choir, harmony, art and music history, aesthetics, acoustics, organology, improvisation, accompanying, chamber music and/or orchestra. They both had more than 15 years of teaching and interpreting experience. They also had at least 10 years of experience as evaluators in music student competitions.

We derived two global indexes of SR. An index of global performance at sight was calculated by the means of the first four variables (tempo maintenance, rhythmic accuracy, pitch accuracy, articulation accuracy). Moreover, another global index of proportion of SR error (SR error) was calculated by dividing the total number of failed notes or rests by the total number of notes and rests written in the score. To obtain this index, the total notes and rests of each score were counted by the evaluators. The raters listened the recordings and marked with lines in a paper copy of the score of each participant the notes failed as a function of rhythm, pitch or articulation, together with repetitions or failed rests. Then, the number of errors was counted and its proportion calculated for each participant and score.

#### Updating Task

We used a Spanish adaptation of the updating task (Carriedo et al., 2016) developed by De Beni and Palladino (2004). This task allowed us to obtain three different indexes – under variable demands of load and suppression – that would correspond to the components of updating executive function described by Ecker et al. (2010): (a) an index of the retrieval and transformation sub-processes (recall of critical items) more related to WMC measures, and (b) two indexes of the substitution sub-process: same lists intrusions, as an index of suppression of information in WM; and previous lists intrusions, as an index of proactive interference or suppression of irrelevant information retrieved from LTM. This task was used in previous research as part of a larger project. For a complete description of the stimulus see Carriedo et al. (2016), and Herrero and Carriedo (2018).

#### Practice Related Skills

We collected information through interviews about the number of years of instrument individual lessons from the beginning up to the present. We also collected information (in hours per week) about the amount of current practice, adding the time of individual and group lessons (chamber music, band or orchestra) and the amount of individual solo practice at home. Data concerning participants under the age of 18 was obtained by their parents.

### Procedure

All participants were tested individually. The order of the tasks was counterbalanced as well as the order of the binary (A) and ternary (B) SR tests. All participants were evaluated at their music schools.

#### SR Tests

All participants performed two consecutive scores. They were instructed to carefully look at the first score (tempo, bar, key signature) for 30 s to immediately perform the score trying not to make breakdowns or repetitions. Once finished, the same procedure was carried out for the second score. All the performances were audio-registered (SONY^®^ IC Recorder ICD-UX71F) to be subsequently analyzed and evaluated by two independent expert musician judges. Inter-rater correlations for all the SR indexes in both binary and ternary tests were significantly high (average *r* = 0.97, *p* = 0.01). To obtain a unique range of punctuations, the values of all the variables ranged from 0 to 10 were divided by 10.

#### Updating Task

The stimulus of the task were auditory presented in a computer. The presentation order of the stimulus and the items was randomized. E-Prime software, version 2.0 (Psychology Software Tools Inc.^2^), was used for randomization and time control (see Carriedo et al., 2016; Herrero and Carriedo, 2018).

## Results

We first tested whether binary and ternary SR tests differed in difficulty as a function of the time bar. Student *t*-test showed that ternary tests were more difficult than binary ones (all *ps* < 0.050). Thus, we decided to analyze binary and ternary measures separately.

In order to analyze how the global index of SR was related to the three indexes of updating (recall of critical items, same-list intrusions and previous-list intrusions) as a function of both, the difficulty of the SR tasks and the demands on memory load and level of suppression, Pearson’s partial correlations controlling for age and practice (accumulated and current) were computed. As shown in Table 2, the global index of SR in both, binary and ternary tasks was significantly related to the recall of the critical items index (retrieval and transformation) only in high memory load and low suppression condition (binary: *r* = 0.22, *p* = 0.012; ternary: *r* = 0.18, *p* < 0.039). In addition, the global index of SR was significantly related to the proportion of same-list intrusions (index of inhibition in WM) in high load and low suppression condition, but in only binary task (*r* = 0.21, *p* < 0.018). No other significant result was found.

**Table 2:** Pearson’s partial correlations between global SR performance and updating indexes as a function of memory lead and level of suppression (controlled age and practice).

		SR_BIN	SR_TERN
		*N* = 131	*N* = 131
Recall	Low load-low suppression	0.16	0.08
	Low load-high suppression	0.07	0.07
	High load-low suppression	**0.22**^∗^	**0.18**^∗^
	High load-high suppression	0.11	0.02
Intrusions same-lists	Low load-low suppression	-0.11	-0.02
	Low load-high suppression	-0.06	-0.03
	High load-low suppression	**-0.21**^∗^	-0.09
	High load-high suppression	-0.13	-0.03
Intrusions previous lists	Low load-low suppression	-0.06	-0.05
	Low load-high suppression	-0.07	-0.12
	High load-low suppression	-0.03	-0.01
	High load-high suppression	-0.02	-0.02


*Significant results are in bold (^∗^*p* < 0.05).*

Taking into account the results of the partial correlations, we analyzed how each specific index of SR could be differently affected by efficiency in the updating sub-processes as a function of the difficulty of the SR task. Thus, we selected those participants more and less efficient in both, the recall of critical items and the proportion of same-list intrusions in high memory load and low suppression condition. Participants who scored above the upper quartile in the recall of critical items were assigned to the “efficient recall” group (*n* = 43), and those who scored below the lower quartile were assigned to the “less efficient recall” group (*n* = 42). In a similar way, participants who scored above the upper quartile in the proportion of same-list intrusions were assigned to the “efficient suppression” group (*n* = 34), and those who scored below lower quartile were assigned to the “less efficient suppression” group (*n* = 40). Importantly, the sample in the case of the measure of expressiveness was reduced to *n* = 21 for the upper quartile and to *n* = 24 to the lower quartile, given that both, elementary-low and elementary-high groups scored zero in this measure. For the same reason, the sample was reduced to *n* = 20 for the upper quartile and to *n* = 22 to the lower quartile.

Multiple mixed ANCOVA 2 × 2, with group (efficient/less efficient) as between-subject factors, and SR task (binary/easy and ternary/difficult) as within-subject factors, and age and practice (accumulated and current) as covariates were carried out for all indexes of SR performance.

**FIGURE 1:**
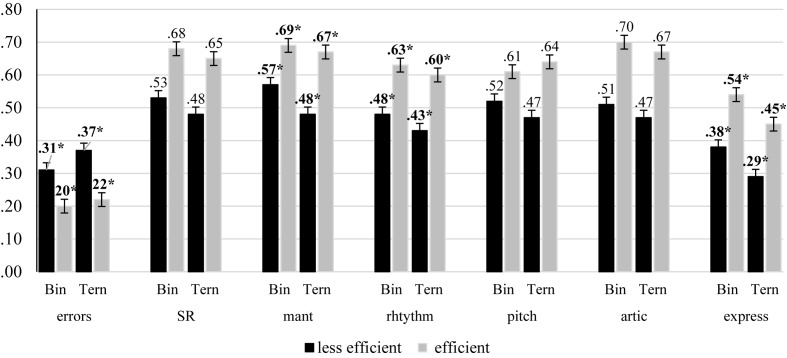
Summary of the means (and standard error bars) for all the indexes evaluated as a function of the difficulty of the task (Bin/Ter), and the efficiency of the participants retrieving and transforming relevant information in WM. Significant differences between efficient and less efficient participants are in bold (^∗^*p* < 0.05).

Table 3 (panels A–G) shows the within and between effects/interactions of all the multiple ANCOVA regarding efficiency in the retrieval and transformation sub-processes. In relation to the global indexes of SR performance, the results showed a significant effect of group in the proportion of SR errors, *F*(1, 80) = 5.07, *p* = 0.027, η*p*^2^ = 0.06, whereas there was no significant effect regarding global performance at sight. In relation to the specific indexes of performance, the results showed a significant effect of group in rhythmic accuracy, *F*(1, 80) = 4.48, *p* = 0.037, η*p*^2^ = 0.05, tempo maintenance, *F*(1, 80) = 4.15, *p* = 0.045, η*p*^2^ = 0.05, and expressiveness, *F*(1, 41) = 5.08, *p* = 0.050, η*p*^2^ = 0.11. In addition, the results showed a significant interaction group × tempo maintenance, *F*(1, 80) = 5.39, *p* = 0.023, η*p*^2^ = 0.06. The analysis of this interaction revealed that whereas efficient participants were not affected by the difficulty of the task, less efficient were (see Figure 1). The results showed no significant effects regarding either pitch and articulation accuracy.

**Table 3:** Within and between effects/interactions of the multiple ANCOVA for all the SR variables as a function of efficiency in the correct recall of critical words.

ANCOVA (covariates Age, Accumulated practice, Current practice)							

*Source of Variation*	*N*	*MS*	*df*	*F*	*p-Value*	η*p*^2^	*Power*
**Panel A. Proportion of errors (Errors)**							
Between subjects							
Age	85	0.01	1	0.17	0.710	0.00	0.07
Accumulated practice	85	0.10	1	0.26	0.612	0.00	0.08
Current practice	85	0.00	1	0.02	0.886	0.00	0.05
Group	**85**	**0.20**	**1**	**5.07**	**0.027**^∗^	**0.06**	**0.61**
Within subjects							
Errors	85	0.01	1	0.64	0.426	0.01	0.12
Errors × Age	85	0.01	1	0.43	0.513	0.01	0.10
Errors × Accumulated practice	85	0.00	1	0.38	0.539	0.01	0.09
Errors × Current practice	85	0.00	1	0.08	0.785	0.00	0.06
Errors × Group	85	0.00	1	0.36	0.553	0.00	0.09
**Panel B. Global SR (SR)**							
Between subjects							
Age	85	0.01	1	0.21	0.648	0.00	0.07
Accumulated practice	85	0.02	1	0.36	0.551	0.00	0.09
Current practice	85	0.02	1	0.15	0.763	0.00	0.06
Group	85	0.23	1	3.77	0.060	0.05	0.48
Within subjects							
SR	85	0.00	1	0.62	0.433	0.01	0.12
SR × Age	85	0.00	1	0.53	0.471	0.01	0.11
SR × Accumulated practice	85	0.00	1	0.60	0.441	0.01	0.12
SR × Current practice	85	0.00	1	0.54	0.452	0.01	0.11
SR × Group	85	0.00	1	0.55	0.461	0.01	0.11
**Panel C. Tempo maintenance (T maint.)**							
Between subjects							
Age	85	0.00	1	0.02	0.891	0.00	0.05
Accumulated practice	85	0.00	1	0.02	0.814	0.00	0.06
Current practice	85	0.00	1	0.03	0.854	0.00	0.05
Group	**85**	**0.29**	**1**	**4.15**	**0.045**^∗^	**0.05**	**0.52**
Within subjects							
T maint.	85	0.00	1	0.05	0.826	0.00	0.06
T maint. × Age	85	0.00	1	0.03	0.870	0.00	0.05
T maint. × Accumulated practice	85	0.00	1	0.11	0.745	0.00	0.06
T maint. × Current practice	85	0.10	1	3.75	0.061	0.04	0.49
T maint. × Group	**85**	**0.15**	**1**	**5.39**	**0.023**^∗^	**0.06**	**0.63**
**Panel D. Rhythmic accuracy (Rhythm)**							
Between subjects							
Age	85	0.04	1	0.48	0.489	0.01	0.11
Accumulated practice	85	0.07	1	0.79	0.378	0.01	0.14
Current practice	85	0.08	1	0.94	0.333	0.01	0.16
Group	**85**	**0.39**	**1**	**4.48**	**0.037**^∗^	**0.05**	**0.55**
Within subjects							
Rhythm	85	0.01	1	0.58	0.450	0.01	0.12
Rhythm × Age	85	0.01	1	0.45	0.506	0.01	0.10
Rhythm × Accumulated practice	85	0.01	1	0.30	0.586	0.00	0.08
Rhythm × Current practice	85	0.02	1	1.08	0.302	0.01	0.18
Rhythm × Group	85	0.01	1	0.52	0.474	0.01	0.11
**Panel E. Pitch accuracy (Pitch)**							
Between subjects							
Age	85	0.06	1	0.46	0.499	0.01	0.10
Accumulated practice	85	0.09	1	0.69	0.410	0.01	0.13
Current practice	85	0.00	1	0.00	0.974	0.00	0.05
Group	85	0.29	1	2.14	0.147	0.03	0.30
Within subjects							
Pitch	85	0.00	1	0.12	0.732	0.00	0.06
Pitch × Age	85	0.00	1	0.10	0.753	0.00	0.06
Pitch × Accumulated practice	85	0.00	1	0.09	0.767	0.00	0.06
Pitch × Current practice	85	0.00	1	0.00	0.994	0.00	0.05
Pitch × Group	85	0.04	1	2.43	0.123	0.03	0.34
**Panel F. Articulation accuracy (Artic.)**							
Between subjects							
Age	85	0.03	1	0.20	0.651	0.00	0.07
Accumulated practice	85	0.06	1	0.39	0.536	0.00	0.07
Current practice	85	0.02	1	0.11	0.746	0.00	0.06
Group	85	0.52	1	3.66	0.061	0.04	0.47
Within subjects							
Artic.	85	0.00	1	0.31	0.579	0.00	0.08
Artic. × Age	85	0.00	1	0.24	0.624	0.00	0.08
Artic. × Accumulated practice	85	0.00	1	0.17	0.685	0.00	0.07
Artic. × Current practice	85	0.01	1	0.45	0.503	0.01	0.10
Artic. × Group	85	0.00	1	0.03	0.862	0.00	0.05
**Panel G. Expressiveness (Express.)**							
Between subjects							
Age	45	0.07	1	1.13	0.303	0.03	0.18
Accumulated practice	45	0.03	1	0.50	0.481	0.01	0.11
Current practice	45	0.02	1	0.24	0.628	0.01	0.08
Group	**45**	**0.31**	**1**	**5.08**	**0.050**^∗^	**0.11**	**0.60**
Within subjects							
Express.	45	0.00	1	0.04	0.854	0.00	0.05
Express. × Age	45	0.00	1	0.16	0.688	0.01	0.07
Express. × Accumulated practice	45	0.00	1	0.23	0.629	0.01	0.08
Express. × Current practice	45	0.01	1	0.49	0.492	0.02	0.10
Express. × Group	45	0.00	1	0.04	0.881	0.00	0.05


*Significant results are in bold (^∗^*p* < 0.05). Group: efficient and less efficient participants; *MS*: main square sum; *df*: degrees of freedom.*

**FIGURE 2:**
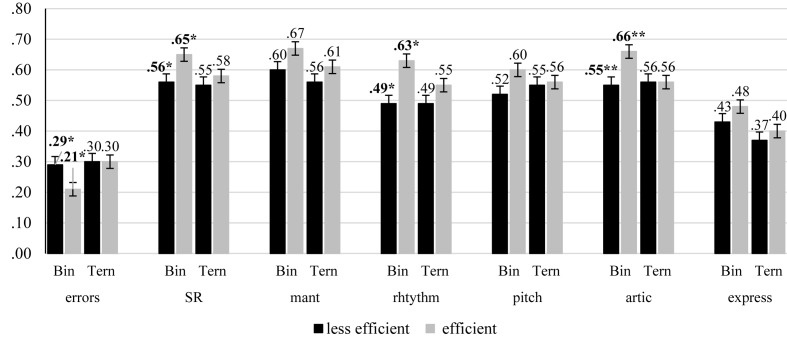
Summary of the means (and standard error bars) for all the indexes evaluated as a function of the difficulty of the task (Bin/Ter), and the efficiency of the participants suppressing irrelevant information from WM. Significant differences between efficient and less efficient participants are in bold (^∗^*p* < 0.05).

Table 4 (panels A to G) shows the within and between effects/interactions of all the multiple ANCOVA regarding efficiency suppressing irrelevant information from WM. In relation to the global indexes of SR performance, the results showed significant interactions in both SR global indexes: group × proportion of SR error, *F*(1, 70) = 3.88, *p* = 0.050, η*p*^2^ = 0.05, group × global SR, *F*(1, 70) = 4.82, *p* = 0.031, η*p*^2^ = 0.06. The analysis of these interactions showed that efficient participants were affected by the difficulty of the SR task whereas those less efficient were not (see Figure 2). Regarding the specific indexes of performance, the results showed a significant interaction group × rhythmic accuracy, *F*(1, 70) = 4.86, *p* = 0.031, η*p*^2^ = 0.07, and group × articulation accuracy, *F*(1, 70) = 11.93, *p* = 0.001, η*p*^2^ = 0.15. As in the case of the global indexes, the analysis of these interactions showed that efficient participants were affected by the difficulty of the SR task whereas those less efficient were not (see Figure 2). There were no significant effects or interactions regarding tempo maintenance, pitch accuracy and expressiveness.

**Table 4:** Within and between effects/interactions of the multiple ANCOVA for all the variables as a function of efficiency in the suppression of no longer relevant words.

ANCOVA (covariates Age, Accumulated practice, Current practice)							

*Source of Variation*	*N*	*MS*	*df*	*F*	*p-Value*	η*p*^2^	*Power*
**Panel A. Proportion of SR error (Errors)**							
Between subjects							
Age	74	0.00	1	0.03	0.875	0.00	0.06
Accumulated practice	74	0.01	1	0.16	0.689	0.00	0.07
Current practice	74	0.01	1	0.17	0.685	0.00	0.07
Group	74	0.06	1	1.53	0.220	0.02	0.23
Within subjects							
Errors	74	0.00	1	0.15	0.699	0.00	0.07
Errors × Age	74	0.01	1	0.27	0.607	0.00	0.08
Errors × Accumulated practice	74	0.01	1	0.23	0.630	0.00	0.08
Errors × Current practice	74	0.01	1	0.25	0.619	0.00	0.08
Errors × Group	**74**	**0.11**	**1**	**3.88**	**0.050**^∗^	**0.05**	**0.50**
**Panel B. Global SR (SR)**							
Between subjects							
Age	74	0.00	1	0.02	0.881	0.00	0.05
Accumulated practice	74	0.03	1	0.23	0.634	0.00	0.08
Current practice	74	0.05	1	0.42	0.521	0.01	0.10
Group	74	0.23	1	1.90	0.172	0.03	0.28
Within subjects							
SR	74	0.00	1	0.01	0.925	0.00	0.05
SR × Age	74	0.00	1	0.05	0.823	0.00	0.06
SR × Accumulated practice	74	0.00	1	0.13	0.723	0.00	0.06
SR × Current practice	74	0.00	1	0.29	0.593	0.00	0.08
SR × Group	**74**	**0.03**	**1**	**4.82**	**0.031**^∗^	**0.06**	**0.58**
**Panel C. Tempo maintenance (T maint.)**							
Between subjects							
Age	74	0.00	1	0.00	0.985	0.00	0.05
Accumulated practice	74	0.00	1	0.04	0.837	0.00	0.06
Current practice	74	0.02	1	0.26	0.613	0.00	0.08
Group	74	0.09	1	1.29	0.260	0.02	0.20
Within subjects							
T maint.	74	0.00	1	0.06	0.815	0.00	0.06
T maint. × Age	74	0.00	1	0.05	0.825	0.00	0.06
T maint. × Accumulated practice	74	0.01	1	0.42	0.518	0.01	0.10
T maint. × Current practice	74	0.10	1	3.60	0.062	0.05	0.47
T maint. × Group	74	0.01	1	0.36	0.552	0.01	0.09
**Panel D. Rhythmic accuracy (Rhythm)**							
Between subjects							
Age	74	0.01	1	0.08	0.784	0.00	0.06
Accumulated practice	74	0.04	1	0.50	0.483	0.01	0.11
Current practice	74	0.21	1	2.37	0.128	0.03	0.33
Group	74	0.26	1	2.90	0.093	0.04	0.39
Within subjects							
Rhythm	74	0.00	1	0.05	0.833	0.00	0.06
Rhythm × Age	74	0.00	1	0.16	0.695	0.00	0.07
Rhythm × Accumulated practice	74	0.01	1	0.33	0.567	0.01	0.09
Rhythm × Current practice	74	0.00	1	0.24	0.627	0.00	0.08
Rhythm × Group	**74**	**0.07**	**1**	**4.86**	**0.031**^∗^	**0.07**	**0.59**
**Panel E. Pitch accuracy (Pitch)**							
Between subjects							
Age	74	0.01	1	0.07	0.797	0.00	0.06
Accumulated practice	74	0.03	1	0.27	0.606	0.00	0.08
Current practice	74	0.05	1	0.40	0.531	0.01	0.10
Group	74	0.15	1	1.15	0.287	0.02	0.19
Within subjects							
Pitch	74	0.03	1	2.34	0.131	0.03	0.33
Pitch × Age	74	0.04	1	2.77	0.102	0.04	0.37
Pitch × Accumulated practice	74	0.04	1	2.93	0.091	0.05	0.38
Pitch × Current practice	74	0.03	1	2.21	0.142	0.03	0.31
Pitch × Group	74	0.04	1	3.04	0.086	0.04	0.40
**Panel F. Articulation accuracy (Artic.)**							
Between subjects							
Age	74	0.00	1	0.00	0.954	0.00	0.05
Accumulated practice	74	0.01	1	0.06	0.808	0.00	0.06
Current practice	74	0.01	1	0.07	0.786	0.00	0.06
Group	74	0.28	1	1.85	0.179	0.03	0.27
Within subjects							
Artic.	74	0.00	1	0.07	0.799	0.00	0.06
Artic. × Age	74	0.00	1	0.13	0.719	0.00	0.07
Artic. × Accumulated practice	74	0.01	1	0.48	0.493	0.01	0.10
Artic. × Current practice	74	0.04	1	3.11	0.082	0.04	0.41
Artic. × Group	**74**	**0.13**	**1**	**11.93**	**0.001**^∗∗^	**0.15**	**0.93**
**Panel G. Expressiveness (Express.)**							
Between subjects							
Age	42	0.16	1	2.70	0.114	0.06	0.36
Accumulated practice	42	0.08	1	1.38	0.253	0.03	0.21
Current practice	42	0.07	1	1.12	0.371	0.01	0.17
Group	42	0.12	1	2.00	0.164	0.04	0.28
Within subjects							
Express.	42	0.06	1	1.69	0.201	0.04	0.25
Express. × Age	42	0.08	1	2.35	0.133	0.05	0.32
Express. × Accumulated practice	42	0.09	1	2.79	0.104	0.06	0.37
Express. × Current practice	42	0.07	1	2.12	0.118	0.05	0.35
Express. × Group	42	0.01	1	0.19	0.668	0.00	0.07


*Significant results are in bold (^∗^*p* < 0.05, ^∗∗^*p* < 0.01). Group: efficient and less efficient participants; *MS*: main square sum; *df*: degrees of freedom.*

## Discussion

We aimed to analyze how the sub-processes of retrieval, transformation and substitution underlying updating in WM were related to SR as a function of the demands on memory load and level of suppression. We also intended to analyze how the efficiency in these updating sub-processes could be a source of individual differences in SR as a function of the difficulty of the SR task and of different indexes of performance.

Regarding the retrieval and transformation sub-processes, the results of the correlational analysis showed that once age and practice were controlled, both SR tasks were significantly related to these sub-processes in high memory load and low suppression condition. These results corroborated our expected relationship and could reflect that SR would rely on memory load more than on interference. Thus, efficient sight readers could have an increased ability to simultaneously maintain and transform into motor movements a greater amount of musical material than less efficient ones, as Meinz and Hambrick (2010) had been already suggested.

The results of the efficiency analysis showed that those participants efficient in retrieving and transforming relevant information in WM committed fewer errors during performance and produced a more expressive execution than less efficient ones. A possible explanation for these results is that the increased ability of our efficient participants could generate improved memory associations between musical fragments. Thus, their range of planning could be enhanced, allowing them to perform without breakdowns or production errors (Drake and Palmer, 2000). In a similar way, the increased range of planning may be responsible for an increased ability to add musical expression to the performance as Lehmann and Kopiez (2009) suggested. Our results also showed that the efficient participants outperformed less efficient ones regarding rhythmic accuracy, which corroborated those results obtained by Kopiez and Lee (2006) with adult expert pianists. However, in our case, the involvement of the retrieval and transformation sub-processes in rhythmic performance were not dependent of the difficulty of the SR tasks as it was in the study of Kopiez and Lee (2006). A possible explanation of these differences could be related to the measurement of rhythmic accuracy in both studies: as metronomic accuracy in the case of Kopiez and Lee or as proportion among notes and rests in our case. In addition, the results showed that efficient participants not only outperformed less efficient ones by maintaining tempo, but neither were their affected by the difficulty of the task, whereas less efficient ones were. It has been suggested that updating in WM could be central to the processing of temporal information due to its involvement in the active maintenance of sequences of events (Carelli et al., 2008). Thus, in ternary tasks – the most demanding ones– those less efficient participants maintaining information in WM had a worse performance keeping a constant speed.

In relation to the substitution sub-process, the results showed a significant relationship to SR in high memory load and low suppression condition, when age and practice were controlled. However, the relationship was significant in only binary tasks. Taking into account the suggested existence of a trade-off effect between maintenance and suppression sub-processes as a function of the availability of attentional resources (e.g., Kane and Engle, 2000; Engle, 2002), However, these attentional resources may be only available in the less demanding SR task, because the difficulty of the ternary tasks could increase memory demands draining the resources to suppress irrelevant information (Lavie et al., 2004). The efficiency analysis also corroborated the lack of resources to inhibit irrelevant information from WM in more demanding SR tasks, which only affected the efficient participants. Specifically, whereas in binary tasks (the easiest ones), the efficient participants committed fewer SR errors than less efficient ones, and outperformed them in rhythmic and articulation accuracy, in ternary tasks (the most difficult task), efficient and less efficient participants performed at about the same level. In this sense, Engle (2002) had been suggested that people with a poorer executive attention would not be affected by the cognitive load to suppress irrelevant information from WM because they would not have attentional resources available even under less demanding conditions, while increasing proactive interference in most demanding ones would decrease performance of the most efficient participants.

Contrary to our hypothesis, the results showed that pitch accuracy did not seem to be affected by the efficiency in both, the retrieval and transformation and the substitution process. A subsequent correlational analysis showed that pitch accuracy was significantly related to accumulated practice, both regarding binary (*r* = 0.18, *p* = 0.037) and ternary (*r* = 0.24, *p* = 0.004) SR tasks. Thus, it seems that pitch accuracy could be more related to instrument practice than for updating abilities during SR. In addition, the results showed that global performance at sight was no affected by efficiency in the retrieval and transformation sub-processes whereas it was affected by efficiency in the substitution one. An explanation for these results is that, because global performance at sight index was a derived measure the significant or no significant effects/interactions of this index would be modulated by the effects/interactions and degree of significance of its components.

Globally, our results corroborated the relevant role of the retrieval and transformation sub-processes in SR, regardless of the difficulty of the SR tasks, which may probably be associated with the implication of WM in this kind of complex activities (Redick et al., 2016). Moreover, our results showed that the substitution sub-process was also involved in performance at sight, but only in low demanding SR tasks. Importantly, our results showed that the updating in WM sub-processes were differently involved in SR according to the different indexes of SR performance. All the updating sub-processes were engaged in SR regarding the proportion of error and rhythmic accuracy, corroborating its reliability as general indexes of SR performance (e.g., Drake and Palmer, 2000; Kopiez and Lee, 2008; Hayward and Gromko, 2009; Wurtz et al., 2009; Mishra, 2014). However, both expressiveness and tempo maintenance seemed to be uniquely driven by efficiency in the retrieval and transformation sub-processes, whereas articulation accuracy relied in the efficiency to suppress irrelevant information from WM. In this sense, expressive indications are associated to a specific musical sequence or fragment to give them full meaning. Moreover, tempo maintenance implies maintaining a constant speed. Thus, it is possible that neither of them require the active suppression of previous information whenever performance continues. However, articulation implies that any musical pattern with concrete rhythm and pitch could appear printed on the score more than once with different articulatory indications. Consequently, the active suppression of previous articulation indications in WM may be necessary to produce a fluent performance.

To conclude, our results support the relevance of updating in WM for SR beyond the effects of age and practice. However, some caution is needed, due to the fact that only two different SR tasks were used. Future research must be done considering a large range of difficulty tasks, especially in order to replicate or to refute the lack of relevance of inhibition in WM in the most difficult tasks. In addition, it is necessary to analyze the contribution of other inhibitory processes such as response-distractor interference, that has been showed to represent an inhibitory factor unrelated to cognitive inhibition measured in our study. Similarly, the role of cognitive flexibility must be also analyzed due to its association with multitasking performance and its involvement in the execution of complex dynamic tasks.

## Ethics Statement

Ethical approval was provided by the bioethics committee of Universidad Nacional de Educación a Distancia (UNED). Parents or guardians of the participants under 18 were informed in writing, and signed the consent of participation of their children in all cases. Young adult participants personally agreed to participate by signing a written informed consent form.

## Author Contributions

LH and NC contributed to the design and implementation of the research, to the analysis of the results, and to the writing of the manuscript.

## Conflict of Interest Statement

The authors declare that the research was conducted in the absence of any commercial or financial relationships that could be construed as a potential conflict of interest.

## Footnotes

1 As Lehmann and Ericsson (1996) suggested, general music performance skills could be highly related to SR in musicians of varied music skill levels, whereas general music performance skills and SR could be dissociated in expert groups in favor of specific SR practice. Thus, practice related skills could refer to both, level of instrument knowledge and specific SR training accumulated.

2 www.pst-net.com/eprime

## References

[B1] Belacchi C. Carretti B. Cornoldi C. (2010). The role of working memory and updating in coloured raven matrices performance in typically developing children. *Eur. J. Cogn. Psychol.* 22 1010–1020. 10.1080/09541440903184617

[B2] Carelli M. G. Forman H. Mäntylä T. (2008). Sense of time and executive functioning in children and adults. *Child Neuropsychol.* 14 372–386. 10.1080/09297040701441411 17852120

[B3] Carretti B. Borella E. Cornoldi C. De Beni R. (2009). Role of working memory in explaining the performance of individuals with specific reading comprehension difficulties: a meta-analysis. *Learn. Individ. Differ.* 19 246–251. 10.1016/j.lindif.2008.10.002

[B4] Carretti B. Cornoldi C. De Beni R. Romanò M. (2005). Updating in working memory: a comparison of good and poor comprehenders. *J. Exp. Child Psychol.* 91 45–66. 10.1016/j.jecp.2005.01.005 15814095

[B5] Carriedo N. Corral A. Montoro P. Herrero L. Rucián M. (2016). Development of the updating executive function: from 7-year-olds to young adults. *Dev. Psychol.* 52 666–678. 10.1037/dev0000091 26882119

[B6] Chiappe P. Siegel L. S. Hasher L. (2000). Working memory, inhib- itory control, and reading disability. *Mem. Cogn.* 28 8–17. 10.3758/BF03211570 10714133

[B7] De Beni R. Palladino P. (2004). Decline in working memory updating through ageing: intrusion error analyses. *Memory* 12 75–89. 10.1080/09658210244000568 15098622

[B8] Drake C. (1993). Reproduction of musical rhythms by children, adult musicians, and adult nonmusicians. *Percept. Psychophys.* 53 25–33. 8433903 10.3758/bf03211712

[B9] Drake C. Palmer C. (2000). Skill acquisition in music performance: relations between planning and temporal control. *Cognition* 74 1–32. 10594308 10.1016/s0010-0277(99)00061-x

[B10] Ecker U. K. Lewandowsky S. Oberauer K. (2014). Removal of information from working memory: a specific updating process. *J. Mem. Lang.* 74 77–90. 10.1016/j.jml.2013.09.003

[B11] Ecker U. K. Lewandowsky S. Oberauer K. Chee A. E. (2010). The components of working memory updating: an experimental decomposition and individual differences. *J. Exp. Psychol. Learn. Mem. Cogn.* 36 170–189. 10.1037/a0017891 20053053

[B12] Elliott C. A. (1982). The relationships among instrumental sight-reading ability and seven selected predictor variables. *J. Res. Music Educ.* 30 5–14.

[B13] Engle R. W. (2002). Working memory capacity as executive attention. *Curr. Direct. Psychol. Sci.* 11 19–23. 10.1111/1467-8721.00160

[B14] Gromko J. E. (2004). Predictors of music sight-reading ability in high school wind players. *J. Res. Music Educ.* 52 6–15. 10.2307/3345521

[B15] Hayward C. M. Gromko J. E. (2009). Relationships among music sight-reading and technical proficiency, spatial visualization, and aural discrimination. *J. Res. Music Educ.* 57 26–36. 10.1177/0022429409332677

[B16] Herrero L. Carriedo N. (2018). Differences in updating processes between musicians and non-musicians from late childhood to adolescence. *Learn. Individ. Differ.* 61 188–195. 10.1016/j.lindif.2017.12.006

[B17] Hodges D. A. (1992). “The acquisition of music reading skills,” in *Handbook of Research in Music Teaching and Learning*, ed. Colwell R. (New York: Schirmer Books), 466–471.

[B18] Just M. A. Carpenter P. A. (1992). A capacity theory of comprehension: individual differences in working memory. *Psychol. Rev.* 99 122–149. 1546114 10.1037/0033-295x.99.1.122

[B19] Kane M. J. Engle R. W. (2000). WM capacity, proactive interference, and divided attention: limits on long-term memory retrieval. *J. Exp. Psychol. Learn. Mem. Cogn.* 26 336–358. 10.10371/0278-7393.26.2.336 10764100

[B20] Kopiez R. Lee J. I. (2006). Towards a dynamic model of skills involved in sight reading music. *Music Educ. Res.* 8 97–120. 10.1080/14613800600570785

[B21] Kopiez R. Lee J. I. (2008). Towards a general model of skills involved in sight reading music. *Music Educ. Res.* 10 41–62. 10.1080/14613800701871363

[B22] Lavie N. Hirst A. de Fockert J. W. Viding E. (2004). Load theory of selective attention and cognitive control. *J. Exp. Psychol. Gen.* 133 339–354. 10.1037/0096-3445.133.3.339 15355143

[B23] Lechuga M. T. Moreno V. Pelegrina S. Gómez-Ariza C. J. Bajo M. T. (2006). Age differences in memory control: evidence from updating and retrieval-practice tasks. *Acta Psychol.* 123 279–298. 10.1016/j.actpsy.2006.01.006 16524555

[B24] Lehmann A. C. Ericsson K. A. (1996). Performance without preparation: structure and acquisition of expert sight-reading and accompanying performance. *Psychomusicology* 15 1–29. 10.1037/h0094082

[B25] Lehmann A. C. Kopiez R. (2009). “Sight-reading,” in *The Oxford Handbook of Music Psychology*, eds Hallam S. Cross I. Thaut M. (Oxford: Oxford University Press), 344–351.

[B26] McPherson G. E. (1994). Factors and abilities influencing sightreading skill in music. *J. Res. Music Educ.* 42 217–231.

[B27] McPherson G. E. Thompson W. F. (1998). Assessing music performance: issues and influences. *Res. Stud. Music Educ.* 10 12–24.

[B28] Meinz E. J. Hambrick D. Z. (2010). Deliberate practice is necessary but not sufficient to explain individual differences in piano sight-reading skill the role of working memory capacity. *Psychol. Sci.* 21 914–919. 10.1177/0956797610373933 20534780

[B29] Mishra J. (2014). Factors related to sight-reading accuracy: a meta-analysis. *J. Res. Music Educ.* 61 452–465. 10.1177/0022429413508585

[B30] Miyake A. Friedman N. P. Emerson M. J. Witzki A. H. Howerter A. Wager T. D. (2000). The unity and diversity of executive functions and their contributions to complex “frontal lobe” tasks: a latent variable analysis. *Cogn. Psychol.* 41 49–100. 10.1006/cogp.1999.0734 10945922

[B31] Morris N. Jones D. M. (1990). Memory updating in working memory: the role of the central executive. *Br. J. Psychol.* 81 111–121.

[B32] Oswald F. L. Hambrick D. Z. Jones L. A. (2007). “Keeping all the plates spinning: understanding and predicting multitasking performance,” in *Learning to Solve Complex Scientific Problems*, ed. Jonassen D. H. (Mahwah, NJ: Erlbaum), 77–97.

[B33] Passolunghi M. C. Pazzaglia F. (2005). A comparison of updating processes in children good or poor in arithmetic word problem-solving. *Learn. Individ. Differ.* 15 257–269. 10.1016/j.lindif.2005.03.001

[B34] Redick T. S. Shipstead Z. Meier M. E. Montroy J. J. Hicks K. L. Unsworth N. (2016). Cognitive predictors of a common multitasking ability: contributions from working memory, attention control, and fluid intelligence. *J. Exp. Psychol. Gen.* 145 1473–1492. 10.1037/xge0000219 27797557

[B35] Sloboda J. A. (1982). “Music performance,” in *The Psychology of Music*, ed. Deutch D. (Cambridge, MA: Academic press), 479–496.

[B36] Smith K. C. Cuddy L. L. (1989). Effects of metric and harmonic rhythm on the detection of pitch alterations in melodic sequences. *J. Exp. Psychol. Hum. Percept. Perform.* 15 457–471. 2527955 10.1037//0096-1523.15.3.457

[B37] St Clair-Thompson H. L. Gathercole S. E. (2006). Executive functions and achievements in school: shifting, updating, inhibition, and working memory. *Q. J. Exp. Psychol.* 59 745–759. 10.1080/17470210500162854 16707360

[B38] Thompson W. B. (1987). Music sight-reading skill in flute players. *J. Gen. Psychol.* 114 345–352.

[B39] Trinity College of London (2003a). *Sight Reading Pieces for Trumpet (Grades 1-8). Sound and Sight*. London: Trinity College of London.

[B40] Trinity College of London (2003b). *Sight Reading Pieces for Violin (Book 1: Initial-Grade 3; Book 2: Grades 4-8). Sound and Sight*. London: Trinity College of London.

[B41] Trinity College of London (2004). *Sight Reading Pieces for Cello (Initial-Grade 8). Sound and Sight*. London: Trinity College of London.

[B42] Trinity College of London (2007a). *Sight Reading Pieces for Bass Clef Brass (Grades 1-8). Sound and Sight*. London: Trinity College of London.

[B43] Trinity College of London (2007b). *Sight Reading Pieces for Clarinet (Book 1: Grades 1-4; Book 2: Grades 5-8). Sound and Sight*. London: Trinity College of London.

[B44] Trinity College of London (2007c). *Sight Reading Pieces for Flute (Book 1: Grades 1-4; Book 2: Grades 5-8). Sound and Sight*. London: Trinity College of London.

[B45] Trinity College of London (2007d). *Sight Reading Pieces for Saxophone (Book 1: Grades 1-4; Book 2: Grades 5-8). Sound and Sight*. London: Trinity College of London.

[B46] Trinity College of London (2007e). *Sight Reading Pieces for Viola (Initial-Grade 8). Sound and Sight*. London: Trinity College of London.

[B47] Trinity College of London (2008). *Sight Reading Pieces for Oboe (Grades 1-8). Sound and Sight*. London: Trinity College of London.

[B48] Trinity College of London (2009a). *Sight Reading Pieces for Double Bass (Initial-Grade 8). Sound and Sight*. London: Trinity College of London.

[B49] Trinity College of London (2009b). *Sight reading pieces for French Horn (Grades 1-8). Sound and Sight*. London: Trinity College of London.

[B50] Waters A. J. Townsend E. Underwood G. (1998). Expertise in musical sight-reading: a study of pianists. *Br. J. Psychol.* 89 123–149.

[B51] Waters A. J. Underwood G. Findlay J. M. (1997). Studying expertise in music reading: use of a pattern-matching paradigm. *Percept. Psychophys.* 59 477–488. 9158323 10.3758/bf03211857

[B52] Wurtz P. Mueri R. M. Wiesendanger M. (2009). Sight-reading of violinists: eye movements anticipate the musical flow. *Exp. Brain Res.* 194 445–450. 10.1007/s00221-009-1719-3 19205680

[B53] Zhukov K. (2017). Experiential (informal/non-formal) practice does not improve sight-reading skills. *Music. Sci.* 21 418–429. 10.1177/1029864916684193 19551491

